# Distance to public transit predicts spatial distribution of dengue virus incidence in Medellín, Colombia

**DOI:** 10.1038/s41598-022-12115-6

**Published:** 2022-05-18

**Authors:** Talya Shragai, Juliana Pérez-Pérez, Marcela del Pilar Quimbayo-Forero, Raúl Rojo, Laura C. Harrington, Guillermo Rúa-Uribe

**Affiliations:** 1 Department of Entomology, Cornell University, Ithaca, NY 14853 USA; 2 Facultad de Medicina, Universidad de Antioquia, 050010 Medellín, Colombia; 3 Centro Administrativo la Alpujarra, Secretaría de Salud de Medellín, 050015 Medellín, Colombia

**Keywords:** Ecological epidemiology, Urban ecology, Entomology

## Abstract

Dengue is a growing global threat in some of the world’s most rapidly growing landscapes. Research shows that urbanization and human movement affect the spatial dynamics and magnitude of dengue outbreaks; however, precise effects of urban growth on dengue are not well understood because of a lack of sufficiently fine-scaled data. We analyzed nine years of address-level dengue case data in Medellin, Colombia during a period of public transit expansion. We correlate changes in the spread and magnitude of localized outbreaks to changes in accessibility and usage of public transit. Locations closer to and with a greater utilization of public transit had greater dengue incidence. This relationship was modulated by socioeconomic status; lower socioeconomic status locations experienced stronger effects of public transit accessibility and usage on dengue incidence. Public transit is a vital urban resource, particularly among low socioeconomic populations. These results highlight the importance of public health services concurrent with urban growth.

## Introduction

Dengue is the most important and fastest growing arboviral disease world-wide. An estimated 50–100 million people are affected each year^1^, and between 1990 and 2020, global burden more than doubled each decade^2^. Available dengue vaccines are not approved in all endemic countries, and require certain safety specifications to administer, and so mitigation primarily relies on mosquito control^3^. Mosquito control resources are limited, and overuse of insecticides causes resistance, forcing many public health programs to target their control efforts in time and space towards areas with an elevated risk of dengue infection. If dengue outbreaks can be identified in the very early stages, efforts can be well-targeted, significantly reducing infection rates^4^. If risk cannot be predicted, control becomes reactive rather than preventative, which can lead to a failure to reduce dengue infection^4^. This importance of early detections has incentivized efforts towards creating accurate dengue outbreak models and risk maps.

Despite the strong incentive and recent advances using machine learning, Bayesian statistics, and other techniques, dengue risk mapping has achieved variable success^5^. Dengue is spatially explicit and highly dependent on the environment and the immunological profile of the human population, creating complex transmission persistence and dispersal patterns^6^. Therefore, dengue transmission shifts dynamically across space and time, complicating the ability to determine reliable predictors. Spatial scale is also a complicating factor. For example, weather is often a primary predictor in dengue models, yet these parameters are often only measured on a homogenous, city-wide scale.

Human mobility has gained increasing recognition as a driver of fine-scale dengue risk. The primary vector of dengue, *Aedes aegypti*, is a short-distance flier^7^, and so the diffusion and spatial variability of dengue across both short and long distances is mediated by human movement^8^. Within a single city, human social networks and daily movement have been shown to predict clusters of dengue infections^9^, and in one study, control via tracing social contacts of infected people effectively reduced dengue^10^. In another study, distance to a metro station predicted the clustering of dengue cases over two epidemic years in Kaohsiung, Taiwan^11^, suggesting that dengue can be tied to hubs of human transport within the space of a city. While lower socioeconomic status has also been tied to dengue incidence in some cases, this effect is highly inconsistent across studies (reviewed in^12^ and^13^). This suggests that it may not be socioeconomic status itself affecting transmission, but other factors that may result from it.

We analyzed dengue cases in Medellín, Colombia during a nine-year period of rapid development of the city’s Metro system. We explored how the construction of public transit infrastructure targeted towards low socioeconomic status regions and the resulting changes in human mobility affected the fine-scale spatial distribution of dengue incidence. Medellín is a perfect test-case to understand the impacts of growing urban infrastructure and public transit on dengue because (1) seasonality is limited, with a stable climate year-round, minimizing noise from climatic drivers of dengue transmission^14^ (2) Medellín has undergone a period of rapid infrastructure growth, including the construction of new public transit lines. This allows for comparison of the spatial structure of dengue before and after the addition of each new line; (3) Medellín has collected probable dengue health care facility case records since 2008, and each case is recorded to the patient’s home address, enabling analysis at a fine spatial scale; (4) Medellín surveyed city-wide human mobility patterns in 2011 and 2016 so we can quantify the use of public transit systems across space to understand its impact on dengue; and (5) Medellin´s neighborhoods are classified based on their socioeconomic strata into six different classes, strata six representing the highest income group, and one the lowest. And there are both areas of high and low socioeconomic status with and without accessible public transit lines throughout the study period.

Medellín is situated in a valley surrounded by mountains. The flat center is primarily industrial and commercial, while more residential neighborhoods are in the steep perimeter. Historically, daily movement to and from the urban industrial city center for low socioeconomic status residents of mountainous parts of the city was limited by lack of accessible transit options and the difficulty of moving on foot^15–17^. Many residents of the high-elevation, high-socioeconomic status regions can travel by personal vehicle or taxi, but for residents of low socioeconomic status regions without the same resources, accessing a job in the industrial center would have required finding a means to traverse up to 600 m in elevation gain. To improve the public transportation system of residents in Medellin, particularly in locations where topography limits the way to move, Medellín Metro system was inaugurated in 1994 with a goal of providing mobility to low socioeconomic status residents of mountainous regions^15–17^. The metro system expanded between 1994 and 2016 to become more accessible and increasingly utilized by larger portions of the city. Medellín has a year-round tropical climate with average temperatures between 21 and 25 °C^14^, and *Ae. aegypti* and recently *Ae. albopictus* have been established across the city^18^. Dengue re-emerged in the Americas in the late twentieth century and it has been regularly diagnosed in Medellin since 1992, with epidemic years recorded in 1998, 2003, and 2007^19^. Between 2002 and 2007, 14,539 cases of dengue were reported^19^. Currently, Medellín is endemic for all four dengue serotypes^20^. Dengue has been a notifiable disease in Colombia since 2008, and in Medellín, all cases diagnosed by a physician that meet the WHO case definition^3^ are reported as probable dengue cases along with each patient’s demographic information and home address (*Medellín Secretaría de Salud*, pers comm).

We conducted a retrospective geospatial analysis of dengue cases in Medellín between 2008 and 2016 to understand the effects of the construction of public transit infrastructure and resulting changes in human mobility and socioeconomic status on fine-scale spatial heterogeneity in dengue risk while accounting for socioeconomic status. We determined if regions of the city that are closer to public transit lines and that have a higher percentage of public transit ridership had higher dengue incidence and analyzed how this effect is modulated by socioeconomic status.

## Results

All analyses were conducted at the spatial level of “SIT zone” (*Zonas Del Sistema Integrado de Transporte*), a zoning metric used by the *Área Metropolitana del Valle de Aburrá* that divides Medellin into 291 spatial units. Over the course of the study period (2008 -2016), the number of reported dengue cases analyzed here varied between 457 and 14,882 analyzed cases per year. Both 2010 and 2016 were epidemic years, with 13, 052 and 14,882 analyzed cases, respectively. In 2008, the metro system consisted of two main lines and two connected arial cable car (*Metrocable*) lines. New lines were added over the study period, reducing the distance to the closest metro line for each zone over time. In 2010, two metro lines (lines A and B) and two arial cable car (*Metrocable*) lines, (lines J and K) were available. In 2011, *Metroplus Linea 1* was added. The *Metroplus* is a bus line with dedicated constructed lanes and stops that connects directly with the metro. In 2012 the *Escaleras electricas* began operating. The *escaleras electricas* are a system of public transit e escalators. They opened on December 28, 2011, but they are analyzed here with the 2012 data. In 2014 the *Metroplus Linea 2* and *Rutas Alimentadoras* were added. The *Metroplus Linea 2* runs along the same route as the *Linea 1* but more than doubles the capacity of the *Metroplus* system. The *Rutas Alimentadoras* are bus lines operated by the city that feed into the metro and *Metroplus* systems and do not run on dedicated lanes. In 2016, a *Tranvia* line and a *Metrocable* line (line H) were added. The *tranvia* is a monorail line.

**Figure 1:**
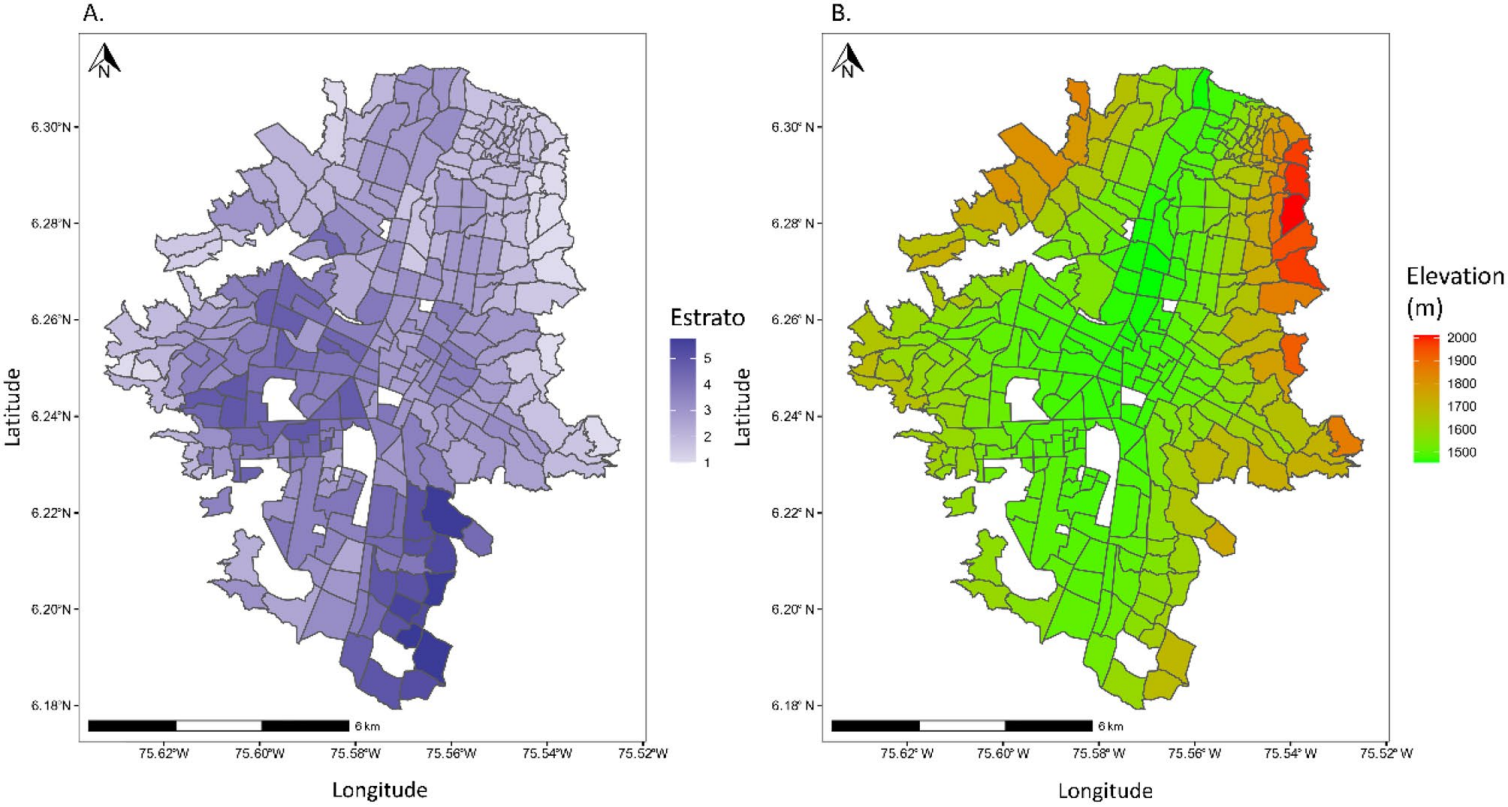
Maps of the transport zones of Medellín showing (**A**) mean socioeconomic status per zone, and (**B**) mean elevation per zone in meters. Blank zones are zones for which there was no data available. Socioeconomic status is measured as *Estrato,* a scale used for socioeconomic classification by the government of Colombia, measuring from 1 (lowest) to 6 (highest). Figure created in R (version 3.4.1) (R Core Team https://www.R-project.org/).

**Figure 2:**
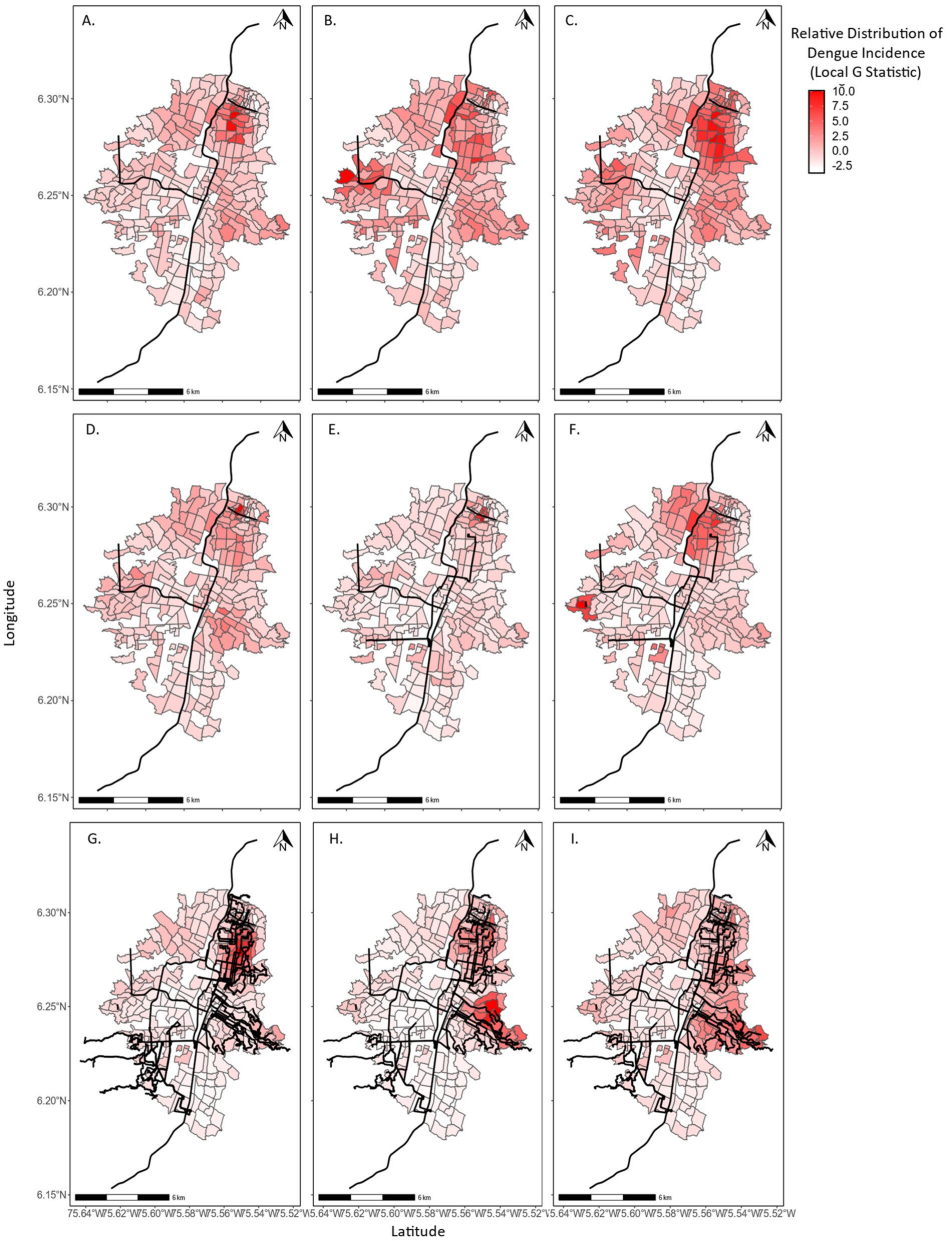
Maps of Medellin, Colombia 2008–2016. Each panel shows the relative distribution of dengue incidence per zone. Relative dengue incidence is shown using the Getis Ord Local G statistic to enable comparisons across years with large differences in the number of total dengue cases. The public transit lines available in each year are shown as black lines for the Metro, *Metroplus, Rutas Alimentadoras,* and the *Escaleras Electricas*. In (**A**) 2008, (**B**) 2009, and (**C**) 2010, two metro lines, Lines (**A**) and (**B**), and two arial cable car (*Metrocable*) lines, Lines J and K were available. In (**D**) 2011, *Metroplus Linea 1* was added. The *Metroplus* is a bus line with dedicated constructed lanes and stops that connects directly with the metro. In (**E**) 2012 the *Escaleras electricas* began operating. The *escaleras electricas* are a system of public transit escelators. Their inauguration was on December 28, 2011, but they are analyzed here with 2012 data. In (**F**) 2013, no new lines were added. In (**G**) 2014 the *Metroplus Linea 2* and *Rutas Alimentadoras* were addded. The *Metroplus Linea 2* runs along the same route as the *Linea 1* but more than doubles the capacity of the *Metroplus* system. The *Rutas Alimentadoras* are bus lines operated by the city that feed into the metro and *Metroplus* systems and do not run on dedicated lanes. In (**H**) 2015, no new lines were added. In (**I**) 2016, a *Tranvia* line and a *Metrocable* line, Line (**H**) were added. The *tranvia* is a monorail line. Figure created in R (version 3.4.1) (R Core Team https://www.R-project.org/).

Between the two years that public transportation was surveyed during the study period (2011 and 2016), the number of respondents using public transportation more than doubled from a median of 5.283% (max = 50.00%, min = 0.00%) of respondents per zone to a median of 11.364% (max = 43.750%, min = 0.00%) of respondents per zone. The socioeconomic status of each zone is shown in Fig. 1. The relative spatial distribution of dengue cases and public transit lines each year is shown in Fig. 2; while our models here analyze dengue incidence (dengue cases per population), Fig. 2 visually represents the distribution of dengue cases as the Getis-Ord Local G Statistic, which is a measure of clustering. Briefly, the Getis-Ord Local G measures the concentration of high or low values for a given study area. This was chosen for the visual representation of dengue cases to enable visual comparison of dengue case spatial distribution across years with varying case counts.

### Dengue incidence, distance to public transit, and socioeconomic status 2008–2016

SIT zones that were closer to public transportation had significantly higher dengue incidence (dengue counts weighted by population) than SIT zones that were farther away from public transit (Estimate = − 0.054, p = 0.0193) (Table 1). Socioeconomic status of a zone alone did not significantly predict dengue incidence (Estimate = − 0.0371, p = 0.340). However, there was a significant positive interaction between income and distance to public transit (Estimate = 0.122, p < 0.0001); the lowest socioeconomic status zones closest to public transit had the highest dengue incidence, while zones with equally low socioeconomic status but farther from public transit had lower dengue incidence. The higher the socioeconomic status of the zone, the less effect distance to transit had on dengue incidence.

**Table 1:** Summary of spatial autoregressive models showing correlation between dengue incidence (dengue counts per population) and distance to nearest transit line, socioeconomic status as measured by *Estrato*, year, and the interaction between *Estrato* and distance to the nearest public transit line in Medellin, Colombia, 2008–2016.

Fixed effects	Dengue incidence	
	Estimate (standard error)	P-value
*Estrato* (scaled)	− 0.037 (0.039)	0.034
Distance to nearest public transit line (scaled)	− 0.054 (0.023)	**0.019**
*Estrato* (scaled): distance (scaled) to nearest public transit line	0.12 (0.017)	** < 0.0001**
**Year**		
2009	− 0.062 (0.048)	0.20
2010	1.71 (0.081)	** < 0.0001**
2011	0.025 (0.048)	0.60
2012	− 0.037 (0.049)	0.45
2013	0.47 (0.051)	** < 0.0001**
2014	0.58 (0.057)	** < 0.0001**
2015	0.68 (0.058)	** < 0.0001**
2016	1.66 (0.082)	** < 0.0001**
Spatial autoregressive coefficient	0.36 (0.024)	** < 0.0001**

Estimates and standard errors are shown. Significant p-values are bolded. *Estrato* and distance to the nearest public transit line have been scaled to enable comparison of effect size. Dengue incidence has been log transformed.

### Dengue incidence, distance to public transit, transit usage, and socioeconomic status in 2011 and 2016

Data was then restricted to 2011 and 2016, the two years that public transit usage was surveyed, and the effects of distance to public transit, public transit usage, and socioeconomic status on reported dengue incidence were analyzed (Table 2). Within these two years, zones closer to public transit had significantly higher reported dengue incidence (Estimate = − 0.136, p = 0.000656) and zones with higher percentage of people reporting using public transit in the previous 24 h had higher reported dengue incidence (Estimate = 0.106, p = 0.0102). There was again no significant main effect of socioeconomic status but there was a significant positive interaction term between distance to public transit and socioeconomic status (Estimate = 0.183, p < 0.0001), as well as a significant positive interaction term between public transit usage and socioeconomic status (Estimate = 0.129, p = 0.000568): low socioeconomic zones with lower ridership or greater distance to transit had a lower dengue incidence than low socioeconomic zones with higher ridership or less distance to transit. 2011 and 2016 were two highly distinct years of dengue infection rates: 2016 was an epidemic year with 14,882 analyzed cases, while 2011 was a post-epidemic year with 513 analyzed cases. Available public transit lines and public transit usage were also very different between these years. In 2011, overall ridership was lower, and most zones did not contain a public transit stop. By 2016, ridership was higher, and most zones contained a public transit stop.

**Table 2:** Summary of spatial autoregressive models for data restricted to 2011 and 2016 showing correlation between dengue incidence (dengue counts per population) and distance to nearest transit line, socioeconomic status as measured by *Estrato*, year, and the interaction between *Estrato* and distance to the nearest public transit line and *Estrato* and percent of survey respondents reporting using public transit in the last 24 h.

Fixed effects	Log(dengue incidence)	
	Estimate (standard error)	P-value
*Estrato* (scaled)	0.052 (0.074)	0.48
Distance to nearest public transit line (scaled)	− 0.14 (0.040)	**0.00037**
Percent of survey respondents using public transit in the last 24 h (scaled)	0.077 (0.041)	0.059
*Estrato* (scaled): distance (scaled) to nearest public transit line	0.18 (0.029)	** < 0.0001**
*Estrato* (scaled): percent of survey respondents using public transit in the last 24 h (scaled)	0.13 (0.037)	** < 0.0001**
**Year**		
2016	1.80 (0.14)	** < 0.0001**
Spatial autoregressive coefficient	0.25 (0.054)	** < 0.0001**

Estimates and standard errors are shown. Significant p-values are bolded. *Estrato,* percent of survey respondents using public transit in the last 24 h, and distance to the nearest public transit line have been scaled to enable comparison of effect size. Dengue incidence has been log transformed.

## Discussion

Our work provides evidence that in Medellín, Colombia, zones that were closer to public transit and had a higher percentage of people reporting using public transit in the last 24 h had higher rates of reported dengue. Furthermore, although living in regions with low socioeconomic status alone did not elevate reported dengue, the combination of low socioeconomic status and high population mobility enabled by public transportation showed to affect dengue incidence.

It is well established that human movement is a major driver of dengue incidence^21,22^, and proximity to and usage of public transport have been previously tied to dengue dynamics^11,23^. However, to our knowledge, this is the first study in which spatial dynamics of dengue incidence are analyzed before and after the construction of new public transit lines. While previous authors have individually studied the relationship between socioeconomic status and dengue risk, we analyzed the interaction of both human movement and socioeconomic status. Low socioeconomic zones of Medellín have high risk conditions for dengue transmission: human density is high, window screens and indoor air conditioning are rare, and there is extensive available habitat for the dengue vector *Ae. aegypti*, as documented by Azoh Barry^24^ and the *Secretaría de Salud* de Medellín (pers comm). However, dengue incidence was not universally higher in low socioeconomic status zones, but rather those zones that were low socioeconomic status and close to transit experienced the highest dengue incidence. It may be that the confluence of higher mobility enabled by public transit and higher risk conditions in lower socioeconomic status zones led to higher incidence. A better understanding how human movement, urban development, and socioeconomic status interact to affect dengue transmission is necessary to fully understand dengue risk.

This work is subject to several important limitations. First, our work shows a relationship between public transportation systems, socioeconomic status, and reported dengue incidence, but does not identify the mechanisms driving observed trends. For example, transportation infrastructure may grow in response to changing population density and as such the two may be intrinsically and historically linked. Alternatively, is possible that in regions with limited mobility, dengue is underreported due to an inaccessibility of medical facilities. Non-severe dengue presents similarly to other febrile diseases that are generally recognized to be self-resolving and non-threatening, and so the incentive to make a difficult trip to a health facility might be low. As public transportation options are built up, more people with dengue might use medical services and case incidence might appear to increase. Another possibility is that because symptomatic dengue is highly associated with secondary infections, areas with historically low incidence and low herd immunity may experience higher secondary attack rates than areas with longstanding incidence, leading to the clusters of incidence observed here. Additionally, dengue cases in Medellin are not laboratory confirmed, but simply meet the WHO criteria for a probable case^3^, and therefore cases may be under or over reported. Other vector-borne diseases such as chikungunya or Zika, or common viral illnesses such as enterovirus infection, influenza, measles, and rubella can be easily confused with dengue when diagnosed clinically. Both Zika and chikungunya were present in Medellin over the study period, and so in particular affect the dengue counts analyzed here. *Ae. aegypti* is a day-time biter and while transmission is likely occurring during the day (reference), it is unknown where the majority of infective bites take place. It is unclear if dengue is increasing due to more infective bites at work, at home via home visits or short distance movements within a community, or in other sites. More research is needed to clarify where dengue transmission takes place. Furthermore, while in this analysis, distance to public transit is measured as a single value for each SIT zone, the SIT zones varied in size, potentially impacting our results. Further research at even finer scales will be necessary to confirm our findings. Finally, it is possible that construction of public transit lines physically alters the landscape in a way that increases transmission by creating more *Ae. aegypti* habitat.

As cities develop, new infrastructure can have unintended consequences on human health. One such consequence might be on the spatial structure of arboviral disease. However, we stress that the conclusion from this study should not be to limit public transit development. The construction of public transportation is one of the most widely recognized methods that governments can use to reliably improve people’s economic conditions. In Medellín, as in other cities, these systems have reduced the commuting time, creating opportunities to residents such as access to jobs, education, public services, and social networks for millions of people, particularly for lower-income communities. Our results highlight the necessity of providing adequate public health services and investing in well-targeted dengue surveillance and outbreak response concurrently with investment to increase human mobility. These results can be used by mosquito control districts to target limited resources, to direct local health departments to provide public health education to citizens and health care providers, and to strengthen health care facilities in coordination with other urban development.

## Materials and methods

### Data

All data was processed and analyzed using R (R Core Team, Version 4.0.3).

Dengue case data were collected and shared by the *Alcaldía de Medellín, Secretaría de Salud*. In Medellin, dengue case surveillance is conducted by public health institutions that classify and report all cases that meet the WHO clinical dengue case criteria for a probable case to Medellin’s *Secretaría de Salud* through SIVIGILA (“*el Sistema Nacional de Vigilancia en Salud Publica)*. All case data were de-identified and aggregated to the SIT Zone level.

Human public transit usage and movement data were collected and shared by the *Área Metropolitana del Valle de Aburrá* for 50–200 respondents per SIT Zone. The “*Encuestas Origen Destino*” (Origen Destination Surveys) were conducted in 2005, 2011, and 2016 and published in 2006, 2012, and 2017, with survey methods described by the *Área Metropolitana del Valle de Aburrá*^25^. Survey respondents include a randomly selected subset of all Medellin residents in each SIT zone regardless of whether they use public transit or not. Survey respondents reported the start and end locations, purpose for travel, and mode of travel for all movement over the last 24 h from the time the survey was administered. Respondents reported all modes of movement, including public transit, private transit, and movement on foot. The results of the survey published in 2017 are published online by the *Área Metropolitana del Valle de Aburrá*^26^, and select data are available through the geodata-Medellin open data portal^27^. The results and data of the survey published in 2012 are not publicly available and were obtained directly from the *Área Metropolitana del Valle de Aburrá.*

The public transit usage survey data were also used to extract socioeconomic data to the SIT zone; surveyors also reported basic demographic data including household *Estrato,* which was averaged per SIT zone to estimate zone socioeconomic status. “*Estrato*” measures socioeconomic status on a scale from 1 (lowest) to 6 (highest). This system is used by the government of Colombia to allocate public services and subsidies (Law 142, 1994). Data from the public transit usage survey were used to extract socioeconomic status data because it is the only location available where the spatial scale of the data matched the spatial scale of the SIT zone.

Data on the location of Medellín public transit lines was downloaded as shape files from the geodata-Medellín open data portal^27^ and subset for each year to the set of transit lines that was available in that year. Data on the opening date of each Medellín public transit line was taken from the Medellín metro website^28^.

Because census data at the zone level were not available for this study and only exists for 2005 and 2018, we used population estimates for each year downloaded from the WorldPop project^29^ and aggregated by SIT zone. The accuracy of WorldPop estimates were checked against available census data for 2005 and 2018 at the *comuna* level, accessed via the geodata- Medellín open data portal^27^.

### Ethical considerations

No human subjects research was conducted. All data used was de-identified, and the analysis was conducted on a database of cases meeting the clinical criteria for dengue with no intervention or modification of biological, physical, psychological, or social variables. All methods were performed in accordance with the relevant guidelines and regulations.

### Data analysis

#### Quantifying public transit usage and distance from nearest transit line

To quantify public transit usage, we determined if each respondent reported using the metro, *metroplus*, or *ruta alimentadora* (supplementary bus route system integrated with the metro system) in the last 24 h. We then calculated the percent of respondents using the public transit system at least once for each SIT zone.

To quantify the distance to the nearest public transit line, we calculated the distance from the center point of each zone to the closest metro, *metroplus*, *tranvía*, *metrocable*, *ruta alimentadora,* or *escalera eléctrica.* This was recalculated for each year, including new transit lines that were added within that year.

### Spatial autoregressive models of dengue incidence

Dengue incidence per year at the level of the SIT zone was modeled using a fixed effects spatial panel model by maximum likelihood (R package splm^30^) as described in^31^. Our fixed effects were socioeconomic status, distance from public transit, a two-way interaction between these factors, and year. To weight dengue cases by population per SIT zone, the model contained a log offset of population per zone per year. Dengue case counts were log transformed after adding one to account for zones with zero dengue cases in a given year. Year was analyzed as a categorical variable to avoid smoothing epidemic years. All continuous variables were scaled to enable comparison of effect size. Because these panel models require balanced data across time, data was truncated to SIT zones that had data for all years available (247 remaining of 291). Spatial dependency was evaluated, and the model was selected using the Hausman specification test and locally robust panel Lagrange Multiplier tests for spatial dependence. Based on a significant Hausman specification test result, which indicates a poor specification of the random effect model, a fixed effect model was chosen. This result is supported by the fact that we had a nearly exhaustive sample of SIT zones in the Medellin metro area. Lagrange multiplier tests were used to determine the most appropriate spatial dependency specifications. Based on the results of the Lagrange multiplier tests, a Spatial Autoregressive (SAR) model was the most appropriate to incorporate spatial dependency; a SAR model considers that the number of dengue cases in a SIT zone depends on the number in neighboring zones.

Because public transit usage was a measurement taken during just two of the study years, we constructed an additional fixed effects spatial panel model by maximum likelihood model of dengue incidence in just 2011 and 2016 that included ridership as an additional predictor variable. Our fixed effects were year, socioeconomic status, distance from public transit, a two-way interaction between socioeconomic status and distance from public transit, percent utilizing public transit, and a two-way interaction between socioeconomic status and percent utilizing public transit. As in our model of all years, the model contained a log offset of population per zone per year and dengue case counts were log transformed after adding one to account for zones with zero dengue cases in a given year, year was analyzed as a categorical variable, and all continuous variables were scaled to enable comparison of effect size. The data was truncated to SIT zones that had data for all years available (251 remaining of 291). We used the same model selection process, and again a fixed effect model was chosen, and based on the results of the Lagrange multiplier tests, a Spatial Autoregressive (SAR) model was determined the most appropriate to incorporate spatial dependency.

## Data Availability

While select data from this study are openly accessible, data on dengue cases and the full public transit use information were obtained according to local laws and policies from the corresponding departments of the *Alcaldía de Medellín.* Therefore, the full curated data set cannot provided as part of this study. Wherever data is available for public access, it has been referenced within the text.
