# Author Correction: Distance to public transit predicts spatial distribution of dengue virus incidence in Medellín, Colombia

**DOI:** 10.1038/s41598-022-15431-z

**Published:** 2022-06-28

**Authors:** Talya Shragai, Juliana Pérez-Pérez, Marcela del Pilar Quimbayo-Forero, Raúl Rojo, Laura C. Harrington, Guillermo Rúa-Uribe

**Affiliations:** 1 Department of Entomology, Cornell University, Ithaca, NY 14853 USA; 2 Facultad de Medicina, Universidad de Antioquia, 050010 Medellín, Colombia; 3 Centro Administrativo la Alpujarra, Secretaría de Salud de Medellín, 050015 Medellín, Colombia

Correction to: *Scientific Reports* 10.1038/s41598-022-12115-6, published online 18 May 2022

The original version of this Article contained an error.

In the Introduction:

“In another study, distance to a metro station predicted the clustering of dengue cases over two epidemic years in Singapore^11^, suggesting that dengue can be tied to hubs of human transport within the space of a city.”

now reads:

“In another study, distance to a metro station predicted the clustering of dengue cases over two epidemic years in Kaohsiung, Taiwan^11^, suggesting that dengue can be tied to hubs of human transport within the space of a city.”

The original Article has been corrected.

